# Global burden of cancer among refugees: A systematic review and meta-analysis

**DOI:** 10.1016/j.jmh.2025.100356

**Published:** 2025-09-20

**Authors:** Fantu Mamo Aragaw, Angela Dawson, Andrew Hayen

**Affiliations:** a School of Public Health, Faculty of Health, University of Technology Sydney, Ultimo, New South Wales, Australia; b Department of Epidemiology and Biostatistics, Institute of Public Health, College of Medicine and Health Sciences, University of Gondar, Gondar, Ethiopia

**Keywords:** Cancer, Global burden, Refugees, Systematic review, Meta-analysis

## Abstract

**Background:**

Refugees are a distinct group of migrants with a variety of cancer risks through cumulative exposures from their country of origin, migration process, and host countries. We conducted a comprehensive systematic synthesis of the epidemiological burden (incidence, prevalence, and mortality) of overall and site specific cancers among refugee populations globally.

**Method:**

We systematically searched in OVID (Medline, Embase), CINAHL, and Scopus for studies reporting cancer prevalence, incidence, or mortality data among refugees globally from database inception to December 15, 2023. We screened for eligible studies using Covidence®. We applied a random-effects model to estimate the pooled proportions of site-specific cancer proportions using R software.

**Result:**

Twenty-nine studies from 12 host countries comprising 561,850 refugees, mainly from Syria and Iraq were included. The pooled proportions for commonly reported site-specific cancer among refugee cancer patients were breast cancer (25.4% (95% CI: 20.3%, 30.4%)), lung cancer (4.8% (95% CI: 3.9%, 5.6%)), leukaemia (16.9% (95% CI: 8.2%, 25.6%)), and central nervous system (CNS) cancer (7.0% (95% CI: 5.3%, 8.7%)) respectively. Studies also reported a higher cervical cancer incidence among female refuges and higher liver cancer mortality among male refugees than non-refugee populations.

**Conclusion:**

Although the existing evidence is limited, this review highlights a notable cancer burden among refugees, with higher incidence and mortality for certain cancers such as cervical and liver cancer among refugees than non-refugees. Targeted practical initiatives in ensuring optimal cancer continuum of care for refugees with cross-disciplinary collaboration are needed.

## Highlights

• Cancer risk varies geographically and across different populations.
• We systematically synthesize the burden of cancer among refugees at a global level.
• There was higher morbidity and mortality rates of specific cancers among refugees than non-refugees.
• Highlights the need for ensuring optimal cancer continuum of care among refugees.

## 1 Introduction

The rising global refugee population resulting from ongoing persecution, political violence, armed conflict, and human rights violations poses a public health challenge that requires health system adaptation (Spiegel et al., 2014; Williamson, 2004; Nikfarid et al., 2020; Trends, 2019). In May 2024, over 120 million people had been forcibly displaced, nearly doubling over the past decade (agency, T.U.R. 2024). About 43.4 million are deemed refugees, who are individuals who have fled their home country due to a well-founded fear of persecution based on their race, religion, nationality, social group, or political opinion and are unable or unwilling to avail themselves of their country's protection (agency.UNHCR, T.U.R.). Refugees are special populations of immigrants as they are forced to leave their native country, whereas immigrants choose to migrate as part of a predetermined process (Alsubhi et al., 2020). Refugees experience of forced displacement, socioeconomic disadvantage, and limited healthcare access increases their likelihood of developing cancer (Erenoğlu and Sözbir, 2020; Altare et al., 2019).

Cancer incidence is increasing worldwide, with a wide geographic distribution, impacting a diverse range of populations (Sung et al., 2021). Globally, around 20 million new cases of cancer and 9.7 million deaths from cancer were reported in 2022 (Bray et al., 2021), and estimates also suggest that around one in every five people develop cancer at some point in their lives (Bray et al., 2024). The World Health Organization (WHO) emphasizes that the increasing global cancer burden disproportionately impacts vulnerable groups, including refugee populations, necessitating urgent action to reduce cancer inequities (organaization, W.h. 2024). Cancer is a critical area of health disparity in under-served populations due to the complex interaction of social and environmental risk factors that contribute to its development, as well as sophisticated diagnostic and treatment strategies (Sze et al., 2015; Wang et al., 2019).

The risk of developing certain cancers varies geographically and migration significantly influences cancer risk (Malagón et al., 2023). Refugees have a set of risk factors for developing cancer which begin in their country of origin, during their migration process and up on arriving in the host country (Spallek et al., 2011).The changing environment resulting from migration and sociocultural changes are accompanied by a transition in cancer risk factors among refugee population (Sullivan et al., 2019).The psychological stress that arises from being forced to leave their country may raise the vulnerability of refugees to a variety of cancers (Palinkas and Pickwell, 1995; Jia et al., 2017). The considerable exposure of refugees to carcinogens and structural risk factors may also raise their risk of developing cancer (Sahloul et al., 2017). Mass displacement of populations can also increase the risk of cancer-causing infections, as it increases the possibility of infectious disease spread (Jawad et al., 2020).

Despite the vulnerabilities, refugees often cannot easily access preventive health services such as cancer screenings, particularly in the early stages of resettlement (Morris et al., 2009; McKeary and Newbold, 2010; Balza et al., 2022). Even refugees resettled in high-income countries with organized cancer screening programs and universal health care have low engagement in cancer screening due to a multitude of barriers, resulting in higher cancer-related mortality rates than non-refugee populations (Tsui and Tanjasiri, 2008; Parajuli et al., 2020; Morrison et al., 2012). Due to this limited access to cancer care services, refugees frequently present at an advanced stage of cancer which leads to complications and high mortality rates (El Saghir et al., 2018).These high levels of morbidity and mortality associated with cancer place a significant burden on both refugees and the healthcare system (Spiegel et al., 2020).

Despite its significant global impact, cancer is the most overlooked NCD among refugees, necessitating a multifaceted prioritization and response (Ilbawi and Slama, 2020; Jubayer et al., 2020; Müller et al., 2018). Disaggregated estimates of refugee cancer morbidity and mortality from global cancer surveillance system such as the GLOBOCAN and cancer registry reports are largely unavailable which contributes to a significant disparity in delivering comprehensive cancer care and its outcomes across these vulnerable populations. Also, most refugees originate from low and middle income countries where cancer registries are not usually well designed to track the epidemiology of cancer (Valsecchi and Steliarova-Foucher, 2008). This evidence gap regarding the pattern of cancer among refugees makes it challenging to effectively address the healthcare needs of refugee populations including cancer prevention programs (Yağcı-Küpeli and Özkan, 2020). Hence, tracking the cancer burden is crucial to find efficient and targeted interventions that meet the distinct needs of refugee populations to improve cancer outcomes among these underserved populations (Mateen et al., 2012).

Existing systematic review assessing non communicable diseases(NCD) prevalence in a forcibly displaced people reported how cancer is overlooked aspect of NCD research in forcibly displaced people without explicitly mentioning the individual studies addressing cancer among these populations (Nishino et al., 2025). Other existing reviews focused on specific refugee group (Al-Oraibi et al., 2022), and refugees are usually studied under the broader migrant category which fails to address their unique experiences. Hence, our study aimed at conducting a comprehensive systematic synthesis of the epidemiological burden (incidence, prevalence, and mortality) of overall and site specific cancers among refugee populations globally. Synthesizing the existing evidence regarding the pattern of cancer in refugees is imperative for informing and prioritizing this issue within global public health policy frameworks. The evidence will provide an insight to anticipate and deliver tailored health interventions that address the unique needs of refugee populations.

## 2 Methods

### 2.1 Protocol and registration

The protocol for this systematic review was prospectively registered in the PROSPERO database (PROSPERO: CRD42024487387). This systematic review and meta-analysis was carried out according to the guidelines provided by the Preferred Reporting Items for Systematic Reviews and Meta-Analysis (PRISMA-P) (Page et al., 2021). Details on the PRISMA checklist are available in the supplementary material (Supplementary Table 1).

### 2.2 Information sources and search strategy

A systematic literature search was conducted from major databases: OVID Medline, OVID Embase, CINAHL, and Scopus. The search was conducted using a combination of medical subject headings (MeSH Terms), and keywords with their synonyms related to cancer and refugees in collaboration with a research librarian at the University of Technology Sydney. The search strategy for each database is found in Supplementary Table 2. Furthermore, we conducted a manual search of the citation lists of the included studies to identify any further potential articles.

### 2.3 Eligibility Criteria

We included all observational studies with prevalence, incidence, or mortality data for any cancer type in the refugee population without restriction to the geographical region, and language of publication. The primary outcome of interest was either prevalence, incidence, or mortality rate of any cancer reported among refugees. Refugees are defined as individuals who have obtained refugee status as a result of being forcefully displaced from their native country due to reasons such as war, political persecution, conflict, or organized violence (Refugees., U.N.H.C.f.). We included peer-reviewed articles published from the conception of databases to December 15, 2023. We excluded commentaries, study protocols, qualitative studies, and studies with non-refugee samples.

### 2.4 Study selection and data extraction

We entered all the studies into EndNote and then uploaded to an online systematic review software tool, Covidence® for management and screening of articles. After removing duplicate entries, we screened articles by titles and abstracts based on predefined eligibility criteria, followed by full-text assessment of the eligible studies for inclusion in the final study. We resolved discrepancies in the study selection by discussion with the reviewers. For each included study, we extracted detailed information about author/year, study country, country of origin of the refugee population, study period, population, data sources, type of study, sample size, and summary of key findings mainly focusing on the epidemiology and stage at diagnosis of cancer among refugees.

### 2.5 Quality of the included studies

We evaluated the quality of the included articles using the Joanna Briggs Institute's (JBI) critical assessment checklist. The JBI quality appraisal tool allows assessment of the methodological quality of a study and evaluates the extent to which a study has addressed the possibility of bias in its design, conduct, and analysis. Each individual study was independently appraised by two reviewers using the applicable JBI critical appraisal checklist, with discrepancies resolved through discussion and consensus. The quality assessment examined each study's methodological rigor, and all studies were retained regardless of quality given the scarcity of evidence and for their valuable insights to design future initiatives in reducing cancer disparities among these vulnerable population groups (Supplementary Table 3).

### 2.6 Data synthesis and statistical analysis

We described the characteristics of the included studies using a table and a narrative synthesis. A random-effects meta-analysis was employed to estimate the pooled proportions of mostly reported site-specific cancer among all refugee cancer patients, and we reported the pooled estimates with 95% CI. The pooled proportions for site-specific cancer proportions were calculated from commonly reported cancer types from the included studies among the overall refugee cancer patients. The random effects meta-analysis was conducted to account for expected heterogeneity among studies utilizing the Der Simonian and Laird estimator with inverse variance weights (DerSimonian and Laird, 1986). The I^2^ statistic was computed to quantify the degree of heterogeneity among studies (Higgins et al., 2003; Huedo-Medina et al., 2006). All statistical analyses were performed using R software version 4.1.2.

## 3 Results

### 3.1 Search results and study characteristics

**Fig. 1:**
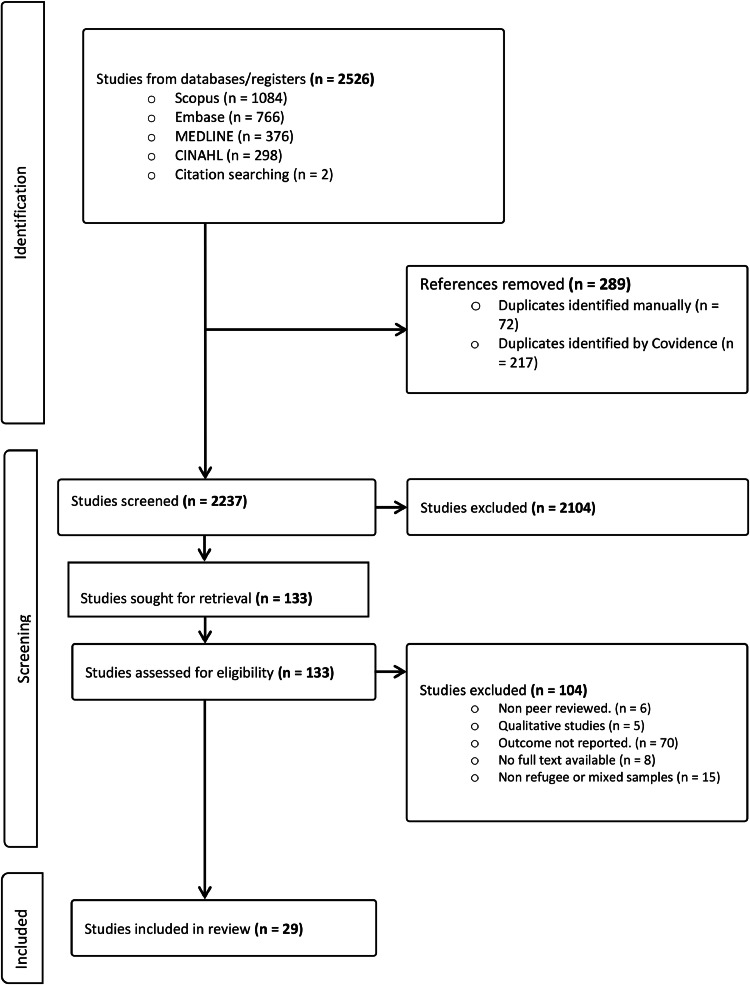
PRISMA Flow chart of the selection of studies included in systematic review and meta-analysis.

Our systematic literature search yielded a total of 2,526 studies. After the initial exclusion of duplicates (289), 2,237 articles were screened based on title and abstract. The remaining 133 articles were reviewed in full text for eligibility, and 29 studies fulfilled the inclusion criteria. The study selection process and the identification of eligible articles were presented using a PRISMA flow diagram (Fig. 1).

The included studies present data on 561,850 refugees from 12 countries with a substantial variation in study sample size ranging from 71 (Yozgat et al., 2023) to 128,962 participants (McDermott et al., 2011). Studies reporting male-to-female distribution show that the male proportion ranged from 13% (Püsküllüoğlu et al., 2023) to 69% (Khan et al., 1997) of the refugee participants. The majority of the studies (26, 90%) were published after 2010, while only 3 were published before 2010 (Khan et al., 1997; DesMeules et al., 2005; Swerdlow, 1991). Data sources used in these studies include electronic or medical health records (Yağcı-Küpeli and Özkan, 2020; Yozgat et al., 2023; Püsküllüoğlu et al., 2023; Khan et al., 1997; DesMeules et al., 2005; Göktaş et al., 2018; Kutluk et al., 2022; Kutluk et al., 2023; Sedef et al., 2021; Yanni et al., 2013; Saleh et al., 2021; Sayan et al., 2022; Soydan et al., 2017; Linton et al., 2020; Yousef et al., 2023; Temi et al., 2017; Eren et al., 2023; Klek et al., 2023), UNHCR records (Spiegel et al., 2014; Spiegel et al., 2020; Mateen et al., 2012; McDermott et al., 2011; Otoukesh et al., 2015), primary data (Ismail et al., 2022; Rehr et al., 2018; Doherty et al., 2020; Bhatta et al., 2015), and cancer registries (Swerdlow, 1991; Rihani et al., 2023). Regarding the geographical distribution of the included studies, about one-third (9, 31%) were conducted in Turkey (Yağcı-Küpeli and Özkan, 2020; Yozgat et al., 2023; Göktaş et al., 2018; Kutluk et al., 2022; Kutluk et al., 2023; Sedef et al., 2021; Sayan et al., 2022; Soydan et al., 2017; Temi et al., 2017), followed by Jordan (7, 24%) (Spiegel et al., 2014; Spiegel et al., 2020; Mateen et al., 2012; Yanni et al., 2013; Yousef et al., 2023; Rehr et al., 2018; Rihani et al., 2023), Lebanon (2, 7%) (Spiegel et al., 2020; Saleh et al., 2021), Bangladesh (2, 7%) (Ismail et al., 2022; Doherty et al., 2020), Canada (2, 7%) (McDermott et al., 2011; DesMeules et al., 2005), Poland (2, 7%) (Püsküllüoğlu et al., 2023; Klek et al., 2023), United States (2, 7%) (Linton et al., 2020; Bhatta et al., 2015), United Kingdom (1, 3%) (Swerdlow, 1991), Syria(1, 3%) (Spiegel et al., 2014), and Iran (1, 3%) (Otoukesh et al., 2015). Most of the refugees countries of origin were from Syria (Spiegel et al., 2014; Spiegel et al., 2020; Yağcı-Küpeli and Özkan, 2020; Yozgat et al., 2023; Göktaş et al., 2018; Kutluk et al., 2022; Kutluk et al., 2023; Sedef et al., 2021; Saleh et al., 2021; Sayan et al., 2022; Yousef et al., 2023; Temi et al., 2017; Rehr et al., 2018; Rihani et al., 2023), Iraq (Spiegel et al., 2014; Spiegel et al., 2020; Mateen et al., 2012; Yozgat et al., 2023; Yanni et al., 2013; Rihani et al., 2023), Afghanistan (Yozgat et al., 2023; Khan et al., 1997; Otoukesh et al., 2015), Ukraine (Klek et al., 2023; Otoukesh et al., 2015), Myanmar (Ismail et al., 2022; Doherty et al., 2020), Somalia (Spiegel et al., 2020), Sudan (Spiegel et al., 2014), Bhutan (Bhatta et al., 2015), Yemen (Rihani et al., 2023), Vietnam (Swerdlow, 1991), and refugees originating from diverse countries (McDermott et al., 2011; DesMeules et al., 2005; Soydan et al., 2017; Linton et al., 2020) (Table 1).

**Table 1:** Characteristics of included studies.

Author/year	Study country	Countries of origin	Study period	Population	Data sources	Type of Study	Sample size	Summary of main findings
A J Swerdlow,1991 (Swerdlow, 1991)	England and Wales	Vietnam	1979-1985	Refugees aged 25 and above	National Health Service Central Register	Prospective cohort study	3327	Mortality in the refugees was greatly increased for cancer of the stomach, cancers of the nasopharynx and liver in males, and peptic ulcer in females. Cancer incidence data showed in addition an excess of cancer of the penis.
McDermott, S., et al. (2011) (McDermott et al., 2011)	Canada	Multicounty	1980- 1990	All age groups of refugees	Refugee Assistance Information System by UNHCR	Retrospective cohort study	128,962	Refugees had a higher SIR than non-refugees for certain cancers such as nasopharyngeal cancer, and liver cancer among male refugees, and refugee women aged 45 to 64 had higher cervical cancer rates than the general population.
DesMeules, M., et al. (2005) (DesMeules et al., 2005)	Canada	Multicounty	1980- 1990	All age group	Canadian Mortality Database	Retrospective cohort study	128,962	Refugee males had an SMR of 4.89(95% CI: 3.29, 6.49) indicating a higher mortality rate for liver cancer than the general Canadian population.
Begül Yağcı-Küpeli & Ayşe Özkan (2020) (Yağcı-Küpeli and Özkan, 2020)	Turkey	Syria	2012- 2019	Refugees aged from 2 months to 17 years	Medical record	Retrospective cohort study	105	The most common cancers reported among Syrian refugee children were Central nervous system tumors (20.9%), Acute lymphoblastic leukemia (17.1%), Neuroblastoma (11.4%), and Wilms tumour (10.5%). Advanced-stage disease and poor compliance with treatment were significantly more frequent in refugees.
Yousef, Y. A., et al. (2023) (Yousef et al., 2023)	Jordan	Syria	2011-2020	Children with Retinoblastoma	Medical record	Retrospective cohort Study	154	Sixteen Syrian refugees (53%) had bilateral Retinoblastoma and Fourteen (47%) had unilateral Retinoblastoma. Refugees were more likely to present with a more advanced stage due to delays in diagnosis and referral.
Linton, N. M., et al. (2020) (Linton et al., 2020)	Washington State	Multicounty	2006 - 2016	All age groups	Worldwide Refugee Admissions Processing System	Retrospective cohort Study	171	Malignant neoplasms were one of the leading causes of death among refugees responsible for one-quarter of deaths. Among the refugee population, the most common sites for neoplasms included the bronchus or lung (16%), brain (11%), and stomach (9%).
Mateen et al.2012 (Mateen et al., 2012)	Jordan	Iraq	2010	All age groups	The Refugee Assistance Information System (RAIS) by UNHCR	Retrospective cohort Study	7642	The number of Iraqi refugees in Jordan diagnosed with primary cancer was 164, accounting for 2.15% (95% CI 1.84, 2.50%) of the refugees seeking health and humanitarian assistance. Among these breast cancer, brain tumours, and genitourinary cancers are the most common types of cancer.
Goktas et al, 2018 (Göktaş et al., 2018)	Turkey	Syria	2012- 2015	All age groups	Electronic medical record	Retrospective Cohort Study	38,243	Out of the total cancer cases, the predominant forms of cancer were breast (28.21%), lymphoid leukaemia (8.11%), colon (6.57%), Hodgkin’s lymphoma (4.87%), brain (3.51%), myeloid leukaemia (3.23%), and non-Hodgkin’s lymphoma (2.80%).
Sayan, M., et al. (2022) (Sayan et al., 2022)	Turkey	Syria	2015 - 2019	Refugees aged 19 to 94 years	Turkish institutional databases	Retrospective cohort study	10,537	Breast cancer (30%) and lung cancer (14%) were the most common diagnoses with up to 68% of patients diagnosed at an advanced stage. One-fifth (20%) of Syrian refugees were identified as noncompliant with radiotherapy.
Yozgat, A. K., et al. (2023) (Yozgat et al., 2023)	Turkey	Multicounty	2011- 2018	Refugee children aged 11 months to 12 years	Health record	Retrospective cohort study	71	The most common cancer diagnosed were acute lymphoblastic leukaemia (16.9%), brain tumours (14%), non-Hodgkin lymphoma (12.6%), and neuroblastoma (11.2%).
Spiegel, P., et al. (2014) (Spiegel et al., 2014)	Jordan and Syria	Iraq Syria Sudan	2010-2012(Jordan), 2009- 2011(Syria)	All age groups	UNHCR’s Exceptional Care Committee record	Retrospective cohort study	2463 (1989 refugees in Jordan and 954 in Syria).	Breast cancer accounted for 23.5% of the total cancer cases, followed by colorectal cancer (12.0%) and soft-tissue cancers (9.8%) in Jordan. In Syria, Breast cancer accounted for 33.1% of cancer cases, followed by leukaemia and other haematological diseases (9.3%), endocrine cancer (7.9%), and colorectal cancer (6.6%).
Spiegel, P. B., et al. (2020) (Spiegel et al., 2020)	Jordan and Lebanon	Syria Iraq Somalia	2015- 2017	All age group	Record review	Retrospective cohort study	733	In Jordan, breast cancer was the most common cancer diagnosis, comprising 95 (33%) of cancer cases. Men primarily presented with genitourinary cancer in Lebanon 40 (21%) of 192 men and with leukaemia in Jordan 22 (18%) of 126 men.
Eren, M. F., et al. (2023) (Eren et al., 2023)	Turkey	Syria	2015- 2019	Refugee male patients	Electronic medical record	Retrospective cohort study	137	Around 64.3% of Syrian refugee patients diagnosed with prostate cancer presented with advanced disease, only 20% received androgen deprivation therapy, and 42% of patients were noncompliant with radiation therapy highlighting significant gaps in adherence to standard care protocols.
Klek, S., et al. (2023) (Klek et al., 2023)	Poland	Ukraine	February 24 to August 24, 2022	All age group	Medical record	Retrospective cohort study	304	Among the 304 cancer cases, breast cancer constituted the highest number of patients with 90 cases (31.5%), followed by colon cancer 15(5.5%), lung cancer 14(4.9%), cervix cancer 14(4.9%), and melanoma 14(4.9%). Of the total cancer cases only 68.4% continued therapy initiated in Ukraine.
Ismail, M., et al. (2022) (Ismail et al., 2022)	Bangladesh	Myanmar	February 2018-July 2018	Forcibly Displaced Myanmar patients aged ≥18 years	Primary data	Cross-sectional study	290	Among the patients 20(6.8%) have malignancy with a proportion of hepatocellular carcinoma 10 (3.4%), Lung cancer 6 (2.1%), stomach cancer 2 (0.7%), and chronic lymphoid leukemia 2 (0.7%).
Rehr, M., et al. (2018) (Rehr et al., 2018)	Jordan	Syria	May 22-June 28, 2016	Adults aged greater than 18 years	Primary data	Cross-sectional household survey	8041	Around 0.6% (0.4–0.7%) of adults were diagnosed with cancer.
Doherty, M., et al. (2020) (Doherty et al., 2020)	Bangladesh	Myanmar	November 20- 24,2017	All age groups	Primary data	Cross-sectional study	156	Around 15(9.6%) of the patients were diagnosed with cancer.
Bhatta, M. P., et al. (2015) (Bhatta et al., 2015)	United States	Bhutan	June- November 2011.	Refugees aged 18 to 65 years old	Primary data	Cross-sectional study	115	The overall prevalence of self-reported cancer of any type was 1.8 % (0.2%–6.4%).
Püsküllüoğlu, M., et al. (2023) (Püsküllüoğlu et al., 2023)	Poland	Ukraine	February 24 - April 8, 2022.	Patients aged 19 to 85 coming for oncology consultation	Clinical records	Cross-sectional study	112	The most frequent diagnosis was breast cancer (43%), followed by gynecological tumours (16%), colorectal cancer (11%), lung cancer (7%), haematological tumours (5%), melanoma (3%), larynx (2%), sarcomas (2%) and thyroid cancer (1%).
Soydan, L., et al. (2017) (Soydan et al., 2017)	Turkey	Multicounty	2013 - 2015	Refugees aged 14 to 87 years	Medical record	Cross-sectional study	1149	Eight (0.7%) of patients who underwent computed tomography of the lung had mass/nodular lesions that are highly suggestive of malignancy.
Otoukesh, S., et al. (2015) (Otoukesh et al., 2015)	Iran	Afghanistan	2005-2010	All age groups	UNHCR record	Cross-sectional study	23 152	Cancer was diagnosed in 3083 patients out of 23,152 referrals. Lymphatic and hematopoietic tissue cancer was the most common cancer in individuals aged 0-17 years, comprising 34.2% of cases, while digestive system cancer was the leading cause of referrals among adults.
Yanni, E. A., et al. (2013) (Yanni et al., 2013)	Jordan	Iraq	2007- 2009	Refugees ≥15 years of age	Medical review	Cross-sectional study	18,990 screened refugees	A total of 97 people had been diagnosed with cancer or were receiving cancer treatment among the registered refugee population.
Saleh, S., et al. (2021) (Saleh et al., 2021)	Lebanon	Syria	July 2018 and January 2020	Adults aged above 18 years old	The ‘Sijilli Electronic Health Records for Refugees database	Cross-sectional study	3255	Among 3255 records of Syrian refugees, 20(0.6%) of refugees were reported to have cancer. Cancer cases were mostly reported among Aleppian refugees. A higher prevalence of cancer was reported among current drinkers (5.9% p = 0.013) than among former alcohol drinkers.
Rihani, R., et al. (2023) (Rihani et al., 2023)	Jordan	Syria, Iraq, and Yemen	2011- 2022	Children with cancer	Cancer Registry	Retrospective cross-sectional study	968	The most common cancer diagnoses among children were leukaemia (41%), lymphoma (25%), solid tumours (24%), and retinoblastoma (6%).
Khan, S. M., et al. (1997) (Khan et al., 1997)	Pakistan	Afghanistan	1990- 1994	Children with biopsy proven cancer	Medical record	Retrospective cross-sectional study	365	Frequently reported cancers in Afghan children were lymphoma (25.2%), lymphoid leukaemia (23%), myeloid leukaemia (12.9%), Wilms tumour (6.3%), retinoblastoma (6.3%), tumours of soft tissue (6%), bone tumours (6%), CNS tumours (2.2%), ovarian tumours (1.6%), and testicular tumours (1%).
Kutluk, T., et al. (2023) (Kutluk et al., 2023)	Turkey	Syria	2011-2020	Children & adult refugees	Health record	Retrospective study	1535 (1114 adults and 421 children)	The most common cancer type was breast cancer 154 (13.8%), leukaemia and multiple myeloma 147 (13.2%), and lymphoma 141 (12.7%) were common among adults. The most frequently diagnosed cancers in children were leukaemia 180 (42.8%), lymphomas 66 (15.7%), and CNS neoplasms 40 (9.5%).
Kutluk et al,2022 (Kutluk et al., 2022)	Turkey	Syria	2005- 2020	Children & adult refugees	Medical records	Retrospective study	268	The most common cancers diagnosed among adults were breast (24.8%), colorectal (10.9%), lung (7.4%), CNS (7.0%), and stomach (5.2%) cancers, while leukaemia (21.1%), lymphoma (21.1%), and CNS cancer (13.2%) were frequently diagnosed among refugee children.
Sedef, A. K., et al. (2021) (Sedef et al., 2021)	Turkey	Syria	2014-2019	Refugees aged 6 to 93 years	Electronic health data/medical records	A Retrospective Observational Study	233	Breast cancer was the most prevalent type, accounting for 35.6%, followed by head and neck cancer at 9.8%. Many patients (47.6%) were diagnosed at advanced stage (stage IV).
Temi, Y. B., et al. (2017) (Temi et al., 2017)	Turkey	Syria	2015- 2017	Refugees aged 18 to 80 years old	Medical record	Retrospective case-series study	134	The most common cancer types were breast (n=57, 42.5%) and gynaecological cancers (n=14, 10.4%).

### 3.2 Prevalence and incidence of cancer among refugee populations

**Fig. 2:**
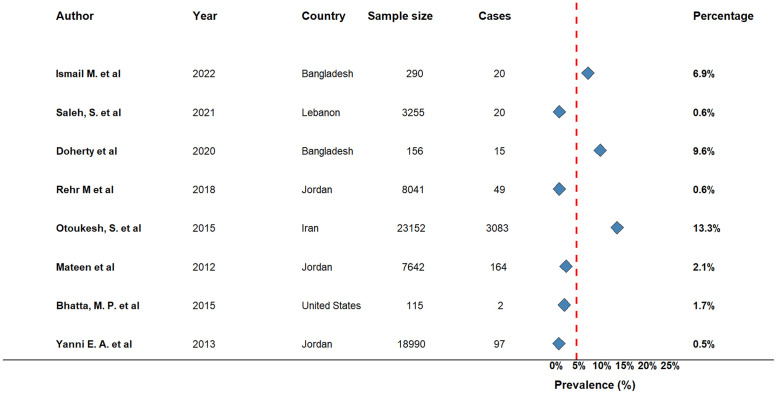
Prevalence of cancer among refugees.

Among 29 included studies, only eight studies (n=61,641) provided data on the prevalence of cancer in the refugee population. The highest reported prevalence of cancer among refugees was 13.3% from a retrospective cross sectional study analyzing cancer profile among Afghan refugees in Iran (Otoukesh et al., 2015), while the lowest prevalence was 0.5% reported from a retrospective review of medical screening data of Iraqi refugees in Jordan (Yanni et al., 2013). A cross-sectional household survey assessing the prevalence of non-communicable diseases revealed the prevalence of cancer among adult Syrian refugees in northern Jordan was 0.6% (Rehr et al., 2018), and another cross sectional study conducted among Rohingya refugees in Bangladesh examining illness related suffering identified 9.6% of participants had cancer (Ismail et al., 2022). A community based cross sectional study examining chronic disease burden among Bhutanese refugee women aged 18–65 years resettled in Northeast Ohio reported an overall cancer prevalence of 1.7% (Bhatta et al., 2015). A secondary analysis of de-identified health records from more than 3,000 Syrian refugees in Lebanon’s Sijilli electronic database identified 0.6% of them had cancer (Saleh et al., 2021). A retrospective study conducted among Iraqi refugees in Jordan revealed that, of the 7,642 individuals registered in the Refugee Assistance Information System (RAIS), 164 were diagnosed with primary cancer (Mateen et al., 2012) (Fig. 2).

Some of the studies reported the stage at diagnosis of cancer among refugee populations. A study conducted among Syrian refugee cancer patients treated in Turkey indicated that about 76.4% and 68% of them were diagnosed at an dvanced stage (III or IV) highlighting a concerning trend of advanced stage cancer diagnosis (Sedef et al., 2021; Sayan et al., 2022). Another comparative study of Syrian refugee children and Turkish children found that 59.8% of patients in Syrian refugee children and 34.8% of Turkish children patients with solid tumours or lymphomas were diagnosed at an advanced stage with a statistically significant higher rate of advanced stage diagnosis among refugees (Yağcı-Küpeli and Özkan, 2020). A study by Temi, Y. Bakkal, et al exploring the characteristics of Syrian refugees with cancer treated in a Turkish hospital revealed that the majority of patients were diagnosed at an advanced stage with 44.8% at stage IV, followed by stage III (32.1%), stage II (18.7%), and stage I (4.5%) (Temi et al., 2017). Almost two third (64.3%) of Syrian refugee men with prostate cancer treated by radiation therapy were diagnosed at an advanced stage, of which 56% diagnosed at stage IV and 9% diagnosed at stage III (Eren et al., 2023). Studies conducted by Kutluk, Tezer, et al across Syrian refugee cancer patients indicated that 43.3% of refugee children and 40.4% of adult refugees were diagnosed at an advanced stage (Kutluk et al., 2022), and the other study reported that 58.7% of patients presented with advanced-stage disease with significant disparities across sex with a higher proportion among men (62.8%) compared to women (53.5%) (Kutluk et al., 2023).

From articles reporting incidence of cancer among refugees, a cohort study of 128,962 refugees and 241,010 non-refugee immigrants in Canada using a linked administrative data reported that older aged refugee women aged 45–64 years had a significantly higher standardized incidence ratio (SIR) of 1.58 (95% CI: 1.06–2.09) of developing cervical cancer compared to the general Canadian population (McDermott et al., 2011).

### 3.3 Distribution of cancer among refugees by cancer type

**Fig. 3:**
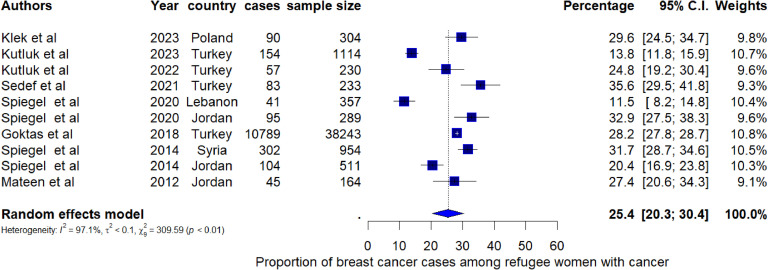
Pooled proportion of breast cancer cases among refugee women with cancer.

**Fig. 4:**
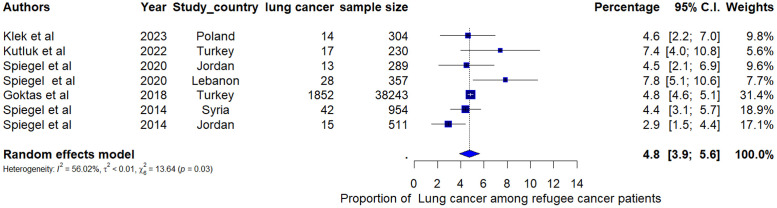
Pooled proportion of lung cancer among refugee cancer patients.

**Fig. 5:**
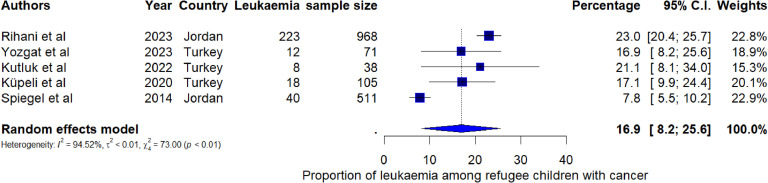
Pooled proportions of leukemia among refugee cancer patients.

Breast cancer was the most common type of cancer among refugee women with a pooled proportion of 25.4% (95% CI: 20.3%, 30.4%, I^2^=97.1%) (Fig. 3). Among the seven studies reporting the proportion of cancer among refugee cancer patients, the pooled proportion of lung cancer was 4.8% (95% CI: 3.9, 5.6; I^2^ = 52.01%) (Fig. 4). Leukaemia was the most reported cancer among children with a pooled proportion of 16.9% (95% CI: 8.2%, 25.6%) (I^2^=94.5%) (Fig. 5). The pooled proportion of CNS cancer was 7.0% (95% CI: 5.3%, 8.7%; I^2^ = 97.8%) (Supplementary Fig. 1). Even though we estimated the pooled proportions of some of the most often reported cancers by most of the studies, there are other types of cancers such as colon cancer, gynaecological cancer, stomach cancer, liver cancer, and Wilms tumour that were reported among refugees.

### 3.4 Cancer-related mortality in refugee populations

Only a few studies have explored cancer-related mortality among refugees. A historical cohort record linkage study conducted among immigrants and the general population in Canada found that male refugees had a substantially higher mortality risk for liver cancer, with a higher standardized mortality ratio (SMR) than the general Canadian population (DesMeules et al., 2005). A study by Linton, N. M., et al examining the mortality rate and causes of death among refugees resettled in Washington state found that malignant neoplasms were one of the leading causes of death, accounting for around one-quarter of refugee deaths (Linton et al., 2020).

## 4 Discussion

Cancer is a significant public health concern among refugee populations. The findings suggest a notable concern of cancer prevalence, incidence, and mortality of certain cancers among refugee population. The magnitude of certain cancer in refugee is higher than non-refugee which may suggest that refugees are a distinct group of migrants with unique experiences and exposures that may increase their vulnerability to various forms of cancer (Jawad et al., 2020). Another possible explanation might be refugees’ face barriers to cancer screening and access to healthcare leading to the rising cancer incidence (Kizilkaya et al., 2022). Nevertheless, understanding the patterns of and risk factors for cancer across refugee groups needs further exploration. Host countries need to track the cancer burden using robust cancer surveillance system with national cancer registries in refugee population to improve cancer outcomes using tailored interventions to refugees' unique healthcare needs.

Besides the reported higher incidence and prevalence of cancer among refugees, most of these refugee cancer cases were diagnosed at an advanced stage which increases the mortality rate as compared to non-refugees (Yağcı-Küpeli and Özkan, 2020; Yozgat et al., 2023; Kutluk et al., 2022; Kutluk et al., 2023). The high rate of advanced stages at the time of diagnosis among refugees emphasizes the need for optimal screening and early diagnosis of cancers in refugee communities to reduce the burden of cancer (Spiegel et al., 2014; Shin et al., 2015; Danaei et al., 2005).

Findings from the review reveals the cancer patterns observed among refugees vary significantly across the refugee populations. Most of the studies attempted to identify prevalent cancer types among refugees and breast cancer has been recognized as the most frequently reported cancer type among refugees among women with a pooled proportion of 26.5% among the total cancer cases (Mateen et al., 2012; Püsküllüoğlu et al., 2023; Göktaş et al., 2018; Kutluk et al., 2022; Kutluk et al., 2023; Sedef et al., 2021; Sayan et al., 2022; Temi et al., 2017; Atag et al., 2023; Alawa et al., 2019). Consistent with the findings, breast cancer ranks as the most prevalent malignancy discovered worldwide, accounting for nearly 24.5% of all cancer cases in women (Sung et al., 2021; Atag et al., 2023; Arnold et al., 2022). The higher rate of breast cancer in refugee populations may be attributed to hereditary and environmental factors, such as poor diet and sedentary lifestyles (Alawa et al., 2020; Al Qadire et al., 2019). Despite the existence of effective cancer screening for breast cancer, most of breast cancer cases among refugees were identified at an advanced stage, with suboptimal treatment, which leads to poor health outcomes (Temi et al., 2017; Alawa et al., 2019; Atag et al., 2023; Abdel-Razeq et al., 2021). To decrease the advanced stage diagnosis and enhance their screening uptake, some studies recommended the integration of breast screening programs into health facilities that serve refugee populations (Mateen et al., 2012).

We found that lung cancer is more commonly reported in male refugees than female refugees (Kutluk et al., 2022; Kutluk et al., 2023). Lung cancer is the most commonly diagnosed cancer and the leading cause of cancer-related deaths among men (Organaizations, W.H. 2024). The substantial smoking rate among men may have influenced the increased prevalence of bronchial and lung cancer as the leading cause of cancer in men compared with women (Kutluk et al., 2022; Kutluk et al., 2023). Additionally, genitourinary cancers, such as bladder cancer, were also reported to be more prevalent in men refugees than in women refugees (Spiegel et al., 2020; Kutluk et al., 2022).

Most of the included studies revealed that refugee children were often diagnosed with leukaemia, lymphoma, and CNS cancers with a pooled proportion of 16.9% for leukaemia, and 6.8% for CNS cancers (Yağcı-Küpeli and Özkan, 2020; Yozgat et al., 2023; Khan et al., 1997; Göktaş et al., 2018; Kutluk et al., 2022; Otoukesh et al., 2015). This finding is similar across other studies conducted in the general child population (Jorjani et al., 2024). Despite the high recovery rate of childhood cancer, refugee children were reported to have lower cancer survival rates compared to non-refugee children (Yozgat et al., 2023; Kebudi et al., 2016). A comparative study conducted to examine the survival rates in children with cancer revealed that Syrian refugee children had a lower survival rate compared to Turkish children (Yağcı-Küpeli and Özkan, 2020). Their challenges in accessing cancer care for optimal treatment and diagnosis is one of the contributing factors to the low survival rates reported among refugee children with cancer (Yozgat et al., 2023). Furthermore, refugees socioeconomic constraints, and suboptimal living conditions, contribute to unfavourable cancer treatment outcomes (Yozgat et al., 2023).

We found a higher incidence of cervical cancer among older-aged refugee women than non-refugees, which may be attributable to refugee women's lower cervical cancer screening uptake (McDermott et al., 2011). Another possible explanation might be the substantial variation in the burden of HPV infection and HPV-related cancers in different regions worldwide (Branda et al., 2024). Higher rates of cervical cancer-associated mortality were also observed among refugees possibly due to the limited healthcare accessibility and the presence of coexisting medical conditions (Fink et al., 2023; Luft et al., 2021). Despite the potential for early detection and treatment of cervical cancers, even refugees living in high-income countries were usually diagnosed at a late stage resulting in suboptimal treatment and higher mortality (Tsui and Tanjasiri, 2008; Temi et al., 2017). Hence, early detection and treatment of cervical cancer are critical in reducing the burden of the disease and its associated mortality.

Notably, the findings from included studies suggest that morbidity and mortality of infection-related cancer such as liver cancer is more common among refugees than host populations (DesMeules et al., 2005). This could be due to refugees’ previous exposure to viral hepatitis in their country of origin, which is strongly associated with an increased risk of liver cancer (DesMeules et al., 2005; Hislop et al., 2007). Similarly, migrants in Europe have a greater incidence of cancers caused by infectious diseases, but a lower incidence of cancers caused by Western lifestyles than the host population (Agyemang and van den Born, 2019). The higher incidence of infection-related cancers in refugee and migrant populations can be attributed to previous exposure to infections in their home countries (McDermott et al., 2011; Agyemang and van den Born, 2019; Walker et al., 2022). Given that infectious diseases contribute substantial and potentially modifiable risk factors for cancer development (de Martel et al., 2020), early prevention through screening and immunization measures upon their arrival of refugees would significantly reduce the risk of infection-related cancers.

About a third of all cancers can be prevented, and an additional third has the potential to be cured by detecting at an early stage (Sahloul et al., 2017; Kanavos, 2006), initiating health screening programs within host country health systems for refugees will reduce the burden of cancer among these vulnerable population groups (Otoukesh et al., 2015). Effective implementation of cancer screening programs necessitates addressing the complex challenges that hinder refugees access to healthcare services (Spiegel et al., 2020). Investing on tailored services that meet refugees' unique health requirements for preventive cancer screening can enhance the health outcomes of refugee communities (Rihani et al., 2023; Anderson et al., 2011).

This is the first study to systematically synthesize the pattern of cancer in refugee populations at a global level. The study's potential limitations including the significant heterogeneity across the included studies to estimate the pooled effects should be carefully considered while interpreting the results. The use of patient-reported diagnoses of cancer in one study may introduce potential bias when estimating the pooled prevalence of overall cancer among refugees. Another limitation is that the population included in the studies may not be representative of the global refugee population. Additionally, we found a paucity of studies assessing burden of cancer among refugee populations underscoring the need for further research to better understand the cancer pattern across refugee groups.

## 5 Conclusion

Although the existing evidence is limited, this review highlights a notable cancer burden among refugees, with higher incidence and mortality for certain cancers such as cervical and liver cancer among refugees than non-refugees. Targeted practical initiatives in ensuring optimal cancer continuum of care for refugees with cross-disciplinary collaboration are needed. Future research and policy reforms need to focus on how to most effectively intervene the cancer disparities to reduce its burden among refugee populations.

## Abbreviation and acronyms

CI: Confidence interval, CNS: central nervous system, JBI: Joanna Briggs Institute, MeSH: Medical Subject Heading, NCDs: noncommunicable diseases, PRISMA-P:Preferred Reporting Items for Systematic Reviews and Meta-Analysis, PROSPERO: International Prospective Register of Systematic Reviews, RAIS: Refugee Assistance Information System, SIR: Standardized incidence ratios, SMR: Standardized mortality ratio, UNHCR: United Nations High Commissioner for Refugees, WHO: World Health Organization.

## Availability of data and materials

The data used to generate the outcomes can be made available upon reasonable request.

## Ethics statement

Not applicable

## Consent for publication

Not applicable.

## Funding

This review was supported by the 10.13039/501100000925National Health and Medical Research Council (NHMRC) [grant number: 2011883] with the project entitled ‘Creating health in a New Home: A Transformative Approach to Build Evidence for Refugee Health across Generations. FMA was supported with the higher degree by research scholarship program from the University of Technology Sydney. The funders had no role in the design and conduct of this review.

## CRediT authorship contribution statement

**Fantu Mamo Aragaw:** Writing – review & editing, Writing – original draft, Visualization, Software, Methodology, Formal analysis, Data curation, Conceptualization. **Angela Dawson:** Writing – review & editing, Visualization, Validation, Supervision, Project administration, Methodology, Investigation, Data curation, Conceptualization. **Andrew Hayen:** Writing – review & editing, Visualization, Validation, Supervision, Software, Methodology, Investigation, Formal analysis, Data curation, Conceptualization.

## Declaration of competing interest

The authors declare that they have no known competing financial interests or personal relationships that could have appeared to influence the work reported in this paper.

## Appendix Supplementary materials

Image, application 1

Image, application 2
