# Supplementary material for: Global burden of cancer among refugees: A systematic review and meta-analysis
Source: J Migr Health. 2025 Sep 20;12:100356. doi: 10.1016/j.jmh.2025.100356 (PMC12508822; doi:10.1016/j.jmh.2025.100356)
Supplement: Supplementary file 1 [file mmc1.pdf]

Authors	Year	Study_country	CNS	sample size	Percentage	95% C.I.	Weights
Kutluk et al	2023	Turkey	40	421	9.5	[6.7; 12.3]	8.9%
Rihani et al	2023	Jordan	2	968	0.2	[0.0; 0.5]	11.6%
Yozgat et al	2023	Turkey	10	71	14.1	[6.0; 22.2]	3.3%
Kutluk et al	2022	Turkey	16	230	7.0	[3.7; 10.2]	8.2%
Sedef et al	2021	Turkey	20	233	8.6	[5.0; 12.2]	7.8%
Küpeli et al	2020	Turkey	22	105	21.0	[13.2; 28.7]	3.5%
Spiegel et al	2020	Jordan	9	289	3.1	[1.1; 5.1]	10.1%
Spiegel et al	2020	Lebanon	47	357	13.2	[9.7; 16.7]	7.9%
Goktas et al	2018	Turkey	1343	38243	3.5	[3.3; 3.7]	11.6%
Spiegel et al	2014	Jordan	37	511	7.2	[5.0; 9.5]	9.7%
Spiegel et al	2014	Syria	55	954	5.8	[4.3; 7.2]	10.7%
Mateen et al	2012	Jordan	15	164	9.1	[4.7; 13.6]	6.6%
**Random effects model**				.	**7.0**	**[5.3; 8.7]**	**100.0%**

Heterogeneity: $I^2 = 97.84\%$, $\tau^2 < 0.01$, $\chi^2_{11} = 509.03$ ($p < 0.01$)

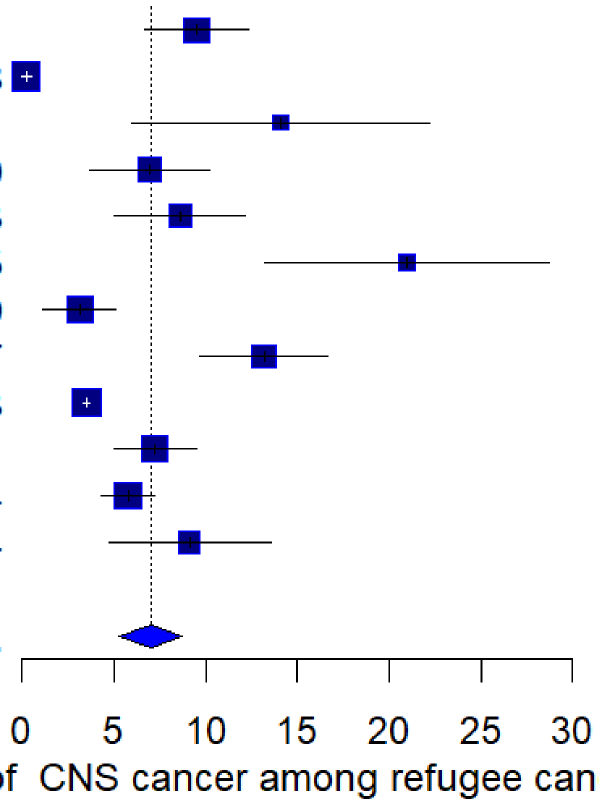

Supplementary Fig 1: Pooled proportion of CNS cancer among refugee cancer patients
