# Supplementary material for: Global burden of cancer among refugees: A systematic review and meta-analysis
Source: J Migr Health. 2025 Sep 20;12:100356. doi: 10.1016/j.jmh.2025.100356 (PMC12508822; doi:10.1016/j.jmh.2025.100356)
Supplement: Supplementary file 2 [file mmc2.docx]

| Section and Topic  **Supplementary Table 1**: PRISMA checklist | Item # | Checklist item | Location where item is reported |
| --- | --- | --- | --- |
| TITLE | | |  |
| Title | 1 | Identify the report as a systematic review. | Page 1 |
| ABSTRACT | | | Page 4 |
| Abstract | 2 | See the PRISMA 2020 for Abstracts checklist. |  |
| INTRODUCTION | | |  |
| Rationale | 3 | Describe the rationale for the review in the context of existing knowledge. | Page 5, 6, &7 |
| Objectives | 4 | Provide an explicit statement of the objective(s) or question(s) the review addresses. | Page 7 |
| METHODS | | |  |
| Eligibility criteria | 5 | Specify the inclusion and exclusion criteria for the review and how studies were grouped for the syntheses. | Page 8 |
| Information sources | 6 | Specify all databases, registers, websites, organisations, reference lists and other sources searched or consulted to identify studies. Specify the date when each source was last searched or consulted. | Page 8 |
| Search strategy | 7 | Present the full search strategies for all databases, registers and websites, including any filters and limits used. | Supplementary file |
| Selection process | 8 | Specify the methods used to decide whether a study met the inclusion criteria of the review, including how many reviewers screened each record and each report retrieved, whether they worked independently, and if applicable, details of automation tools used in the process. | Page 9 |
| Data collection process | 9 | Specify the methods used to collect data from reports, including how many reviewers collected data from each report, whether they worked independently, any processes for obtaining or confirming data from study investigators, and if applicable, details of automation tools used in the process. | Page 9 |
| Data items | 10a | List and define all outcomes for which data were sought. Specify whether all results that were compatible with each outcome domain in each study were sought (e.g. for all measures, time points, analyses), and if not, the methods used to decide which results to collect. | NA |
|  | 10b | List and define all other variables for which data were sought (e.g. participant and intervention characteristics, funding sources). Describe any assumptions made about any missing or unclear information. | NA |
| Study risk of bias assessment | 11 | Specify the methods used to assess risk of bias in the included studies, including details of the tool(s) used, how many reviewers assessed each study and whether they worked independently, and if applicable, details of automation tools used in the process. | Page 9 |
| Effect measures | 12 | Specify for each outcome the effect measure(s) (e.g. risk ratio, mean difference) used in the synthesis or presentation of results. | Page 9 |
| Synthesis methods | 13a | Describe the processes used to decide which studies were eligible for each synthesis (e.g. tabulating the study intervention characteristics and comparing against the planned groups for each synthesis (item #5)). | Page 9 |
|  | 13b | Describe any methods required to prepare the data for presentation or synthesis, such as handling of missing summary statistics, or data conversions. | NA |
|  | 13c | Describe any methods used to tabulate or visually display results of individual studies and syntheses. | Page 11 |
|  | 13d | Describe any methods used to synthesize results and provide a rationale for the choice(s). If meta-analysis was performed, describe the model(s), method(s) to identify the presence and extent of statistical heterogeneity, and software package(s) used. | Page 11 & 12 |
|  | 13e | Describe any methods used to explore possible causes of heterogeneity among study results (e.g. subgroup analysis, meta-regression). | NA |
|  | 13f | Describe any sensitivity analyses conducted to assess robustness of the synthesized results. | NA |
| Reporting bias assessment | 14 | Describe any methods used to assess risk of bias due to missing results in a synthesis (arising from reporting biases). | NA |
| Certainty assessment | 15 | Describe any methods used to assess certainty (or confidence) in the body of evidence for an outcome. | Page 11, Figures |
| RESULTS | | |  |
| Study selection | 16a | Describe the results of the search and selection process, from the number of records identified in the search to the number of studies included in the review, ideally using a flow diagram. | Page 10, Figures |
|  | 16b | Cite studies that might appear to meet the inclusion criteria, but which were excluded, and explain why they were excluded. |  |
| Study characteristics | 17 | Cite each included study and present its characteristics. | Table 1 |
| Risk of bias in studies | 18 | Present assessments of risk of bias for each included study. | Supplementary file |
| Results of individual studies | 19 | For all outcomes, present, for each study: (a) summary statistics for each group (where appropriate) and (b) an effect estimate and its precision (e.g. confidence/credible interval), ideally using structured tables or plots. | Figures |
| Results of syntheses | 20a | For each synthesis, briefly summarise the characteristics and risk of bias among contributing studies. | Table 1 |
|  | 20b | Present results of all statistical syntheses conducted. If meta-analysis was done, present for each the summary estimate and its precision (e.g. confidence/credible interval) and measures of statistical heterogeneity. If comparing groups, describe the direction of the effect. | Page 11 and 12 |
|  | 20c | Present results of all investigations of possible causes of heterogeneity among study results. | NA |
|  | 20d | Present results of all sensitivity analyses conducted to assess the robustness of the synthesized results. | NA |
| Reporting biases | 21 | Present assessments of risk of bias due to missing results (arising from reporting biases) for each synthesis assessed. | NA |
| Certainty of evidence | 22 | Present assessments of certainty (or confidence) in the body of evidence for each outcome assessed. | Page 11 |
| DISCUSSION | | |  |
| Discussion | 23a | Provide a general interpretation of the results in the context of other evidence. | Page 12-15 |
|  | 23b | Discuss any limitations of the evidence included in the review. | Page 15 |
|  | 23c | Discuss any limitations of the review processes used. | Page 15 |
|  | 23d | Discuss implications of the results for practice, policy, and future research. | Page 16 |
| OTHER INFORMATION | | |  |
| Registration and protocol | 24a | Provide registration information for the review, including register name and registration number, or state that the review was not registered. | Page 8 |
|  | 24b | Indicate where the review protocol can be accessed, or state that a protocol was not prepared. | Page 8 |
|  | 24c | Describe and explain any amendments to information provided at registration or in the protocol. | - |
| Support | 25 | Describe sources of financial or non-financial support for the review, and the role of the funders or sponsors in the review. | Page 16 |
| Competing interests | 26 | Declare any competing interests of review authors. | Page 16 |
| Availability of data, code and other materials | 27 | Report which of the following are publicly available and where they can be found: template data collection forms; data extracted from included studies; data used for all analyses; analytic code; any other materials used in the review. | Page 16 |

**Supplementary Table 2: Search strategy for the study global burden of cancer among refugees: A systematic review and meta-analysis**

| **No** | **Search databases** | **Search terms** | **Search results** |
| --- | --- | --- | --- |
| 1 | Ovid MEDLINE | cancer*.mp. **OR** exp Carcinoma/ or carcinoma*.mp. or exp Carcinoma in Situ/ **OR** neoplas*.mp. **OR** malignancy.mp. or exp Neoplasms/ **OR** tumo?r*.mp. or exp Carcinoid Tumor/ **OR** malignant*.mp. **OR** sarcoma.mp. or exp Sarcoma/ **OR** teratoma*.mp. **OR** exp Lymphoma/ or lymphoma*.mp. or Lymphoma, Non-Hodgkin/ **OR** leukemia*.mp. or exp Leukemia/ or Leukemia, Myeloid/ **OR** myeloma*.mp.  **AND**  refugee*.mp. or exp Refugees/ **OR** "refugee camp*".mp. or Refugee Camps/ **OR** "forcibly displaced person*".mp. **OR** "forcibly displaced people*".mp. **OR** "forced migrant*".mp. | 376 |
| 2 | Ovid Embase | advanced cancer/ or cancer*.mp.  **OR** carcinoma*.mp. or exp carcinoma/ **OR** exp neoplasm/ or neoplas*.mp. **OR** malignancy.mp. **OR** tumo?r*.mp. **OR** malignant*.mp. or malignant neoplasm/ or malignant teratoma/ **OR** sarcoma/ or sarcoma.mp. **OR** teratoma*.mp. or malignant teratoma/ or teratoma/ **OR**  lymphoma/ or non-Hodgkin lymphoma/ or lymphoma*.mp **OR** exp leukemia/ or exp myeloid leukemia/ or leukemia*.mp **OR** myeloma*.mp. or exp myeloma/ **OR** adenocarcinoma*.mp. or exp adenocarcinoma/  **AND**  Refugees.mp. **OR** exp refugee/ or refugee*.mp. **OR** "refugee camp*".mp. or exp refugee camp/ **OR** "forcibly displaced person*".mp **OR** "forcibly displaced people*".mp **OR** "forced migrant*".mp. | 766 |
| 3 | Scopus | ( TITLE-ABS-KEY ( cancer* **OR** carcinoma* **OR** "Carcinoma in Situ" **OR** neoplas* **OR** malignancy **OR** neoplasms **OR** tumor* **OR** "Carcinoid Tumor" **OR** malignant* **OR** sarcoma **OR** teratoma* **OR** lymphoma* **OR** "Non-Hodgkin lymphoma" **OR** leukemia* **OR** myeloma* ) AND TITLE-ABS-KEY ( refugee* **OR** "refugee camp*" **OR** "forcibly displaced person*" **OR** "forcibly displaced people*" **OR** "forced migrant*" ) ) | 1084 |
| 4 | CINHAL | "cancer" **OR** "Carcinoma" **OR** "neoplasm" **OR** "malignancy" **OR** "tumo?r" **OR** "malignant"  **OR** (MH "Sarcoma+") **OR** "teratoma" **OR** (MH "Lymphoma+") **OR** "Non-Hodgkin lymphoma" **OR** "leukemia" **OR** "myeloma" **AND** ("refugee" **OR** (MH "Refugees+") **OR** (MH "Refugee Camps")  **OR** "forced migrant" | 298 |

**Supplementary Table 3: JBI quality appraisal of included studies.**

The table below displays the response for each question of the critical appraisal checklist for cohort studies, prevalence studies and case series studies respectively with a response denoted as 'yes (Y)', 'no (N)', 'unclear (U)', or 'not applicable (NA).

| Studies | Were the two groups similar and recruited from the same population? | Were the exposures measured similarly to assign people to both exposed and unexposed groups? | Was the exposure measured in a valid and reliable way? | Were confounding factors identified? | Were strategies to deal with confounding factors stated? | Were the groups/participants free of the outcome at the start of the study (or at the moment of exposure)? | Were the outcomes measured in a valid and reliable way? | Was the follow up time reported and sufficient to be long enough for outcomes to occur? | Was follow up complete, and it not, were the reasons to loss to follow up described and explored? | Were strategies to address incomplete follow up utilized? | Was appropriate statistical analysis used? |
| --- | --- | --- | --- | --- | --- | --- | --- | --- | --- | --- | --- |
| McDermott, S., et al. (2011)([1](#_ENREF_1)) | N | Y | Y | N | N | Y | Y | Y | N | Y | Y |
| DesMeules, M., et al. (2005)([2](#_ENREF_2)) | Y | Y | Y | Y | Y | Y | Y | Y | N | Y | Y |
| A J Swerdlow,1991([3](#_ENREF_3)) | NA | NA | Y | Y | N | Y | Y | Y | N | N | Y |
| Studies | Was the sample frame appropriate to address the target population? | Were study participants sampled in an appropriate way? | Was the sample size adequate? | Were the study subjects and the setting described in detail? | Was the data analysis conducted with sufficient coverage of the identified sample? | Were valid methods used for the identification of the condition? | Was the condition measured in a standard, reliable way for all participants? | Was there appropriate statistical analysis? | Was the response rate adequate, and if not, was the low response rate managed appropriately? | | |
| Kutluk, T., et al. (2023)([4](#_ENREF_4)) | Y | Y | Y | Y | Y | Y | Y | Y | NA |  | |
| Rihani, R., et al. (2023)([5](#_ENREF_5)) | Y | Y | Y | Y | Y | Y | Y | Y | NA |  |  |
| Püsküllüoğlu, M., et al. (2023)([6](#_ENREF_6)) | Y | N | N | Y | Y | Y | Y | Y | U |  |  |
| Yousef, Y. A., et al. (2023)([7](#_ENREF_7)) | Y | N | N | Y | Y | Y | Y | Y | U |  |  |
| Yozgat, A. K., et al. (2023) ([8](#_ENREF_8)) | Y | Y | N | Y | Y | Y | Y | Y | NA |  |  |
| Eren, M. F., et al. (2023)([9](#_ENREF_9)) | Y | Y | U | Y | Y | Y | Y | Y | NA |  |  |
| Klek, S., et al. (2023)([10](#_ENREF_10)) | Y | Y | Y | Y | Y | Y | Y | Y | NA |  |  |
| Kutluk et al,2022([11](#_ENREF_11)) | Y | Y | Y | Y | Y | Y | Y | Y | NA |  |  |
| Ismail, M., et al. (2022) ([12](#_ENREF_12)) | Y | Y | Y | Y | Y | Y | Y | Y | Y |  |  |
| Sayan, M., et al. (2022)([13](#_ENREF_13)) | Y | N | Y | Y | Y | Y | Y | Y | NA |  |  |
| Saleh, S., et al. (2021)([14](#_ENREF_14)) | Y | Y | Y | Y | Y | Y | Y | Y | U |  |  |
| Sedef, A. K., et al. (2021)([15](#_ENREF_15)) | Y | Y | Y | Y | Y | Y | Y | Y | NA |  |  |
| Linton, N. M., et al. (2020)([16](#_ENREF_16)) | Y | Y | Y | Y | Y | Y | Y | Y | NA |  |  |
| Spiegel, P. B., et al. (2020)([17](#_ENREF_17)) | U | U | Y | Y | Y | Y | Y | Y | NA |  |  |
| Doherty, M., et al. (2020)([18](#_ENREF_18)) | Y | Y | Y | Y | Y | Y | Y | Y | U |  |  |
| Begül Yağcı-Küpeli & Ayşe Özkan (2020)([19](#_ENREF_19)) | Y | N | N | Y | Y | Y | Y | Y | NA |  |  |
| Goktas et al, 2018([20](#_ENREF_20)) | Y | Y | Y | Y | Y | Y | Y | Y | NA |  |  |
| Rehr, M., et al. (2018)([21](#_ENREF_21)) | Y | Y | Y | Y | Y | Y | Y | Y | Y |  |  |
| Soydan, L., et al. (2017)([22](#_ENREF_22)) | Y | Y | Y | Y | Y | N | N | Y | U |  |  |
| Otoukesh, S., et al. (2015)([23](#_ENREF_23)) | Y | Y | Y | Y | Y | U | Y | Y | NA |  |  |
| Yanni, E. A., et al. (2013).([24](#_ENREF_24)) | Y | Y | Y | Y | Y | Y | Y | Y | U |  |  |
| Bhatta, M. P., et al. (2015)([25](#_ENREF_25)) | Y | Y | Y | Y | Y | N | N | Y | U |  |  |
| Spiegel, P., et al. (2014)([26](#_ENREF_26)) | U | U | Y | Y | Y | Y | Y | Y | NA |  |  |
| Mateen et al.2012([27](#_ENREF_27)) | Y | Y | Y | Y | Y | Y | U | Y | NA |  |  |
| Khan, S. M., et al. (1997)([28](#_ENREF_28)) | Y | Y | Y | Y | Y | Y | Y | Y | NA |  |  |
| Studies | Were there clear criteria for inclusion in the case series? | Was the condition measured in a standard, reliable way for all participants included in the case series? | Were valid methods used for identification of the condition for all participants included in the case series? | Did the case series have consecutive inclusion of participants? | Did the case series have complete inclusion of participants? | Was there clear reporting of the demographics of the participants in the study? | Was there clear reporting of clinical information of the participants? | Were the outcomes or follow up results of cases clearly reported? | Was there clear reporting of the presenting site(s)/clinic(s) demographic information? | Was statistical analysis appropriate? | |
| Temi, Y. B., et al. (2017)([29](#_ENREF_29)) | Y | Y | Y | N | Y | Y | Y | Y | Y | Y | |

**References**

1. McDermott S, DesMeules M, Lewis R, Gold J, Payne J, Lafrance B, et al. Cancer incidence among Canadian immigrants, 1980–1998: results from a national cohort study. Journal of immigrant and minority health. 2011;13:15-26.

2. DesMeules M, Gold J, McDermott S, Cao Z, Payne J, Lafrance B, et al. Disparities in mortality patterns among Canadian immigrants and refugees, 1980–1998: results of a national cohort study. Journal of Immigrant and Minority Health. 2005;7:221-32.

3. Swerdlow AJ. Mortality and cancer incidence in Vietnamese refugees in England and Wales: a follow-up study. International Journal of Epidemiology. 1991;20(1):13-9.

4. Kutluk T, Şahin B, Kirazlı M, Ahmed F, Aydın S, Çınkır HY, et al. Clinical Characteristics and Outcomes of Cancer Cases Among Syrian Refugees From Southern Turkey. JAMA network open. 2023;6(5):e2312903-e.

5. Rihani R, Jeha S, Nababteh M, Rodriguez-Galindo C, Mansour A, Sultan I. The burden and scope of childhood cancer in displaced patients in Jordan: The King Hussein Cancer Center and Foundation Experience. Frontiers in Oncology. 2023;13:1112788.

6. Püsküllüoğlu M, Grela-Wojewoda A, Szczubiałka G, Zemełka T, Lompart J, Sałek-Zań A, et al. Cancer care for Ukrainian refugees during the first 6 weeks of 2022 Russian invasion–An experience of a cancer reference centre in Poland. European Journal of Cancer. 2023;178:234-42.

7. Yousef YA, Abu Salim QF, Mohammad M, Jaradat I, Mehyar M, AlJabari R, et al. Presentation and management outcomes of Retinoblastoma among Syrian refugees in Jordan. Frontiers in Oncology. 2023;12:1056963.

8. Yozgat AK, Özyörük D, Emir S, Demir A, Erdem AY, Aker CB, et al. Evaluation of Leukemia and Solid Tumors in Refugee Children in Turkey: A Tertiary Center Experience: Refugee Children with Cancer in Turkey. The Journal of Pediatric Academy. 2023;4(2):62-6.

9. Eren MF, Kilic SS, Eren AA, Kaplan SO, Teke F, Kutuk T, et al. Radiation therapy for prostate cancer in Syrian refugees: facing the need for change. Frontiers in Public Health. 2023;11:1172864.

10. Klek S, Chrobak-Kasprzyk K, Machnicka K, Kret K, Litewka A, Kantor N, et al. Cancer Care to Ukrainian War Refugees in Poland. JAMA Network Open. 2023;6(7):e2321967-e.

11. Kutluk T, Koç M, Öner İ, Babalıoğlu İ, Kirazlı M, Aydın S, et al. Cancer among syrian refugees living in Konya Province, Turkey. Conflict and Health. 2022;16(1):3.

12. Ismail M, Hussain MF, Al Hasan MA, Kamal AM, Rahman M, Hasan MJ. Health problems among forcibly displaced Myanmar Nationals (fdmns) admitted to the Medicine Ward of Cox's Bazar Medical College Hospital. Journal of migration and health. 2022;6:100123.

13. Sayan M, Eren MF, Kilic SS, Kotek A, Kaplan SO, Duran O, et al. Utilization of radiation therapy and predictors of noncompliance among Syrian refugees in Turkey. BMC cancer. 2022;22(1):1-7.

14. Saleh S, Abdouni L, Dimassi H, Nabulsi D, Harb R, Jammoul Z, et al. Prevalence of non-communicable diseases and associated medication use among Syrian refugees in Lebanon: an analysis of country-wide data from the Sijilli electronic health records database. Conflict and Health. 2021;15(1):1-12.

15. Sedef AK, Temi YB, Bahceci A, Sedef AM. Retrospective case series study on basic epidemiological characteristics of the cancer diagnosed Syrian refugees in south of Turkey. EJMI. 2021;5(4):496-9.

16. Linton NM, DeBolt C, Newman LP, Tasslimi A, Matheson J. Mortality rate and causes of death among refugees resettled in Washington State, 2006–2016. Journal of immigrant and minority health. 2020;22:3-9.

17. Spiegel PB, Cheaib JG, Aziz SA, Abrahim O, Woodman M, Khalifa A, et al. Cancer in Syrian refugees in Jordan and Lebanon between 2015 and 2017. The Lancet Oncology. 2020;21(5):e280-e91.

18. Doherty M, Power L, Petrova M, Gunn S, Powell R, Coghlan R, et al. Illness-related suffering and need for palliative care in Rohingya refugees and caregivers in Bangladesh: a cross-sectional study. PLoS medicine. 2020;17(3):e1003011.

19. Yağcı-Küpeli B, Özkan A. Syrian and Turkish children with cancer: a comparison on survival and associated factors. Pediatric hematology and oncology. 2020;37(8):707-16.

20. Göktaş B, Yılmaz S, Gönenç İM, Akbulut Y, Sözüer A. Cancer incidence among Syrian refugees in Turkey, 2012–2015. Journal of International Migration and Integration. 2018;19:253-8.

21. Rehr M, Shoaib M, Ellithy S, Okour S, Ariti C, Ait-Bouziad I, et al. Prevalence of non-communicable diseases and access to care among non-camp Syrian refugees in northern Jordan. Conflict and health. 2018;12:1-14.

22. Soydan L, Demir AA, Tunaci A. Frequency of abnormal pulmonary computed tomography findings in asylum seeking refugees in Turkey. International Health. 2017;9(2):118-23.

23. Otoukesh S, Mojtahedzadeh M, Figlin RA, Rosenfelt FP, Behazin A, Sherzai D, et al. Literature review and profile of cancer diseases among Afghan refugees in Iran: referrals in six years of displacement. Medical Science Monitor: International Medical Journal of Experimental and Clinical Research. 2015;21:3622.

24. Yanni EA, Naoum M, Odeh N, Han P, Coleman M, Burke H. The health profile and chronic diseases comorbidities of US-bound Iraqi refugees screened by the International Organization for Migration in Jordan: 2007–2009. Journal of immigrant and minority health. 2013;15:1-9.

25. Bhatta MP, Shakya S, Assad L, Zullo MD. Chronic disease burden among Bhutanese refugee women aged 18–65 years resettled in Northeast Ohio, United States, 2008–2011. Journal of immigrant and minority health. 2015;17:1169-76.

26. Spiegel P, Khalifa A, Mateen FJ. Cancer in refugees in Jordan and Syria between 2009 and 2012: challenges and the way forward in humanitarian emergencies. The lancet oncology. 2014;15(7):e290-e7.

27. Mateen FJ, Carone M, Al-Saedy H, Nyce S, Mutuerandu T, Ghosn J, et al. Cancer diagnoses in Iraqi refugees. Acta Oncologica. 2012;51(7):950-1.

28. Khan SM, Gillani J, Nasreen S, Zai S. Pediatric tumors in north west Pakistan and Afghan refugees. Pediatric hematology and oncology. 1997;14(3):267-72.

29. Temi YB, Sedef AM, Gokcay S, Coskun H, Kaplan SO, Ozkul O, et al. A study on basic demographic and disease characteristics of cancer-diagnosed Syrian refugees treated in the border city of Turkey, Sanliurfa; a hospital-based retrospective case series study. Breast cancer. 2017;57:42.5.
